# Stem Cell Therapies for Ischemic Stroke: A Systematic Review

**DOI:** 10.7759/cureus.13139

**Published:** 2021-02-04

**Authors:** Abba Musa Abdullahi, Ibrahim Muhammad Abdullahi, Shah T Sarmast, Arpita Bhriguvanshi

**Affiliations:** 1 Life Sciences, University of South Wales, Newport, GBR; 2 Biomedical Sciences, University of Nottingham, Nottingham, GBR; 3 Neurology, California Institute of Behavioral Neurosciences and Psychology, Fairfield, USA; 4 Pediatrics, Oregon Health & Science University, Portland, USA

**Keywords:** stem cells, ischemic stroke, therapy, transplantation, regeneration, repair

## Abstract

Stroke is one of the leading causes of death and disability worldwide. It is associated with a high economic burden, causing an increasing demand for highly effective, curative, and long-lasting therapies. Stem cells are unique human cells that have the capacity for developing into specialized cell types with the potential for facilitating regeneration and repair of damaged tissues. Therefore, many preclinical studies have shown the feasibility, safety, and efficacy of stem cell-based therapies; however, the evidence is still inadequate for their therapeutic use in humans. We employed a systematic approach to search published data from 2000 to 2020 on five main databases: PubMed, PubMed Central, Google Scholar, ScienceDirect, and Medline. Two research registries were also searched: the Cochrane Registry and clinicaltrial.gov. Data was collected after applying inclusion and exclusion criteria and studies were appraised critically. Both Medical Subject Headings (MeSH) and regular keyword search strategies were employed. The findings of this study are in line with previously reported studies in which stem cell-based therapies were found to be relatively safe, feasible, and effective.

## Introduction and background

Stroke was defined by the World Health Organization (WHO) in 1970 as "rapidly developed clinical signs of focal (or global) disturbance of cerebral function, lasting more than 24 hours or leading to death, with no apparent cause other than of vascular origin" [1]. There are two main classes of stroke: ischemic stroke and hemorrhagic stroke. Ischemic stroke occurs as a result of occlusion of cerebral blood vessels, usually caused by either thrombosis or embolism. Hemorrhagic stroke occurs due to the rupture of cerebral blood vessels or aneurysms [2]. Ischemic stroke is the most common type, constituting about 85% and occurs in stages: initially, there is an occlusion of a blood vessel (an artery), which causes decreased blood flow and consequently, ischemia, which, if not reversed, will immediately lead to infarction, an irreversible cellular death, and the area is referred to as “ischemic core”, and the surrounding area of surviving neurons is called “ischemic penumbra”, which could be recovered if blood flow is restored within the first three hours or six hours in some instances. This time period is referred to as the therapeutic window [3,4].

The functional recovery is mainly determined by the capacity to rescue the ischemic penumbra and therefore interventions for early reperfusion like revascularization and thrombolysis were the main therapies towards salvaging the ischemic penumbra [5]. However, due to the narrow therapeutic window, only a minority (about 5%) of stroke patients benefit from these therapies [3]. Therefore, many therapies with a wide window of opportunity have been investigated to explore new therapeutic options. Stem cell therapies have been recognized as potential neuroregenerative therapies for stroke patients, which can be used effectively in both acute and chronic phases of stroke [6,7]. These novel therapeutic strategies function via multiple mechanisms involving angiogenesis, neurogenesis, synaptic plasticity, and immune modulation, which results in both functional and structural regeneration of the brain tissues [3,6]. Stem cells can be classified based on their origin as either embryonic (obtained from embryos prior to implantation) or adult (somatic) stem cells obtained from matured differentiated tissues such as the bone marrow. Additionally, stem cells can be categorized based on their differentiation capacity as either totipotent, pluripotent, or multipotent [8]. There are many types of stem cells that are currently used in the clinical trials of patients with ischemic stroke that demonstrate high efficacy and a good safety profile and hence are suitable for use in humans. The common stem cells used are bone marrow mononuclear cells consisting of mesenchymal stem cells (MSCs) and hematopoietic stem cells (HSCs), neural stem cells, embryonic stem cells, and human-induced pluripotent stem cell (iPSC) [9]. However, the most attractive stem cell is the bone marrow mononuclear cells due to its rapid regeneration and its composition of mesenchymal, hematopoietic, and endothelial progenitor cells [10].

Many systematic reviews and meta-analyses have demonstrated favorable clinical outcomes in stroke patients following stem cell transplantation, based on preclinical studies; however, there is not enough clinical evidence to support its use in humans [4]. Therefore, the purpose of this study is to describe the feasibility, safety, and efficacy of different stem cell therapies in patients with ischemic stroke.

## Review

1. Methods

1.1. Study Design and Protocol

We conducted a systematic review of published literature involving a synthesis of both graphical and narrative information. The protocol employed for this study was based on the "Preferred Reporting Items for Systematic Reviews and Meta-Analyses (PRISMA)" guidelines [11]. The review protocol was prepared by the research team and then reviewed by two experts in the field who were not part of the research team.

1.2. Sources of Data Collection and Search Strategy

An electronic search of published studies was conducted to identify relevant articles from 2000 to March 2020 on the following databases: PubMed, ScienceDirect, PubMed Central, Google Scholar, and Medline. Also, two major trial research registries were searched: Cochrane Central Register (Cochrane Library 2020, Issue 3) and clinicaltrial.gov (www.clinicaltrials.gov). The reference lists of the included articles were also searched for relevant studies. Additionally, a search for unpublished literature was performed by contacting two renowned experts in the field. Both MeSH and regular keyword search strategies were used for the identification of the relevant articles. Mesh strategy was mainly used for searching PubMed while regular keywords were employed mainly for other databases.

*1.3. Search Content* The followings keywords/terms were used for the identification of relevant articles: “stem cell therapy” AND “ischemic stroke” AND “human” AND “clinical trials”, “stem cell transplantation” AND “ischemic stroke” AND “human” AND “clinical trials”, “bone marrow transplantation” AND “ischemic stroke” AND “human” AND “clinical trials”, “stem cell therapy” AND “stroke” AND “human” AND “clinical trials”.

*1.4. Inclusion and Exclusion Criteria* We applied the following inclusion criteria for the data collection: 1) Studies done in the last 20 years, from the year 2000 to March 2020. 2) Studies done exclusively on patients with ischemic stroke. 3) Studies done only on humans. 4) Clinical trial studies including both randomized and non-randomized open-label, single-arm, and comparative studies. 5) Studies where the bone marrow was used to treat ischemic stroke in any phase of the disease (acute, sub-acute, or chronic), irrespective of the source of the cell (autograft, allograft, or xenograft), route of the cell administration (intravenous, intra-arterial, intrathecal, intracerebral or subcutaneous), and dosage. 6) Studies done globally. 7) Studies done in English or translated into English. We excluded any trial that combined the assessment of two or more therapies in addition to stem cell therapies unless if it was the conventional stroke therapy. We also excluded any study that was not in English or translated into English.

*1.5. Population* We included patients with ischemic stroke in any phase of the disease, from acute, sub-acute to chronic, and at any time after the onset of the index stroke regardless of their age, gender, or country.

*1.6. Intervention* The interventions we included in this review were stem cell-based interventions with any form of stem cell transplantation irrespective of the source of the cell (autograft, allograft, or xenograft), route of the cell administration (intravenous, intra-arterial, intrathecal, intracerebral, or subcutaneous), and dosage.

*1.7. Comparison* Patients with ischemic stroke who received stem cell transplantation in the included controlled trials were compared with the groups that either received placebo or conventional stroke management.

*1.8. Outcome* The primary outcomes of interest were assessed after a minimum of six-month follow-up using measures of effectiveness with validated international scales for neurologic impairment [measured by the National Institutes of Health Stroke Scale (NIHSS); 0-42; higher = worse], disability [measured by the modified Rankin Scale (mRS); 0-6; higher = worse], and dependency or activities of daily living [measured by the Barthel Index (BI); 0-100; higher = better]. The secondary outcomes were the post-procedural safety outcomes, and we evaluated the following: death, infection, stroke recurrence, neoplasm, seizures, pain, fever, headache, and hemorrhagic transformation of stroke.

*1.9. Data Extraction* We extracted our data from the included study using standard data extraction form and the information extracted included the following factors: study authors, year of publication, country of study, sample size and study population demographics, recruitment period, phase of the disease, source and type of stem cell transplantation, route of administration, the timing of stem cell transplantation, and outcome data in the intervention group and the follow-up period. Elements of the study designs like randomization, open-label, blinding, single-arm, control, treatment allocation, intervention, and outcomes in the controlled groups were also recorded. Differences between authors were resolved by discussion. Authors were contacted via email for any missing information when there was a need.

*1.10. Risks of Bias Assessments* The risk of bias was determined based on the following domains: random sequence generation, allocation concealment, patient blind and care provider blind, intention-to-treat analysis, outcome assessor blind, incomplete outcome data, selective outcome reporting, and other biases [12].

1.11. Quality Assessment

The Critical Appraisal Skills Programme (CASP) checklist for assessing randomized control trials (2018) was used for assessing the validity of the included randomized control trial studies while the Newcastle-Ottawa Scale (NOS) was used for assessing the quality of non-randomized studies. Low-quality papers were excluded and only high and moderate-quality papers were included in the study.

2. Results

**Figure 1:**
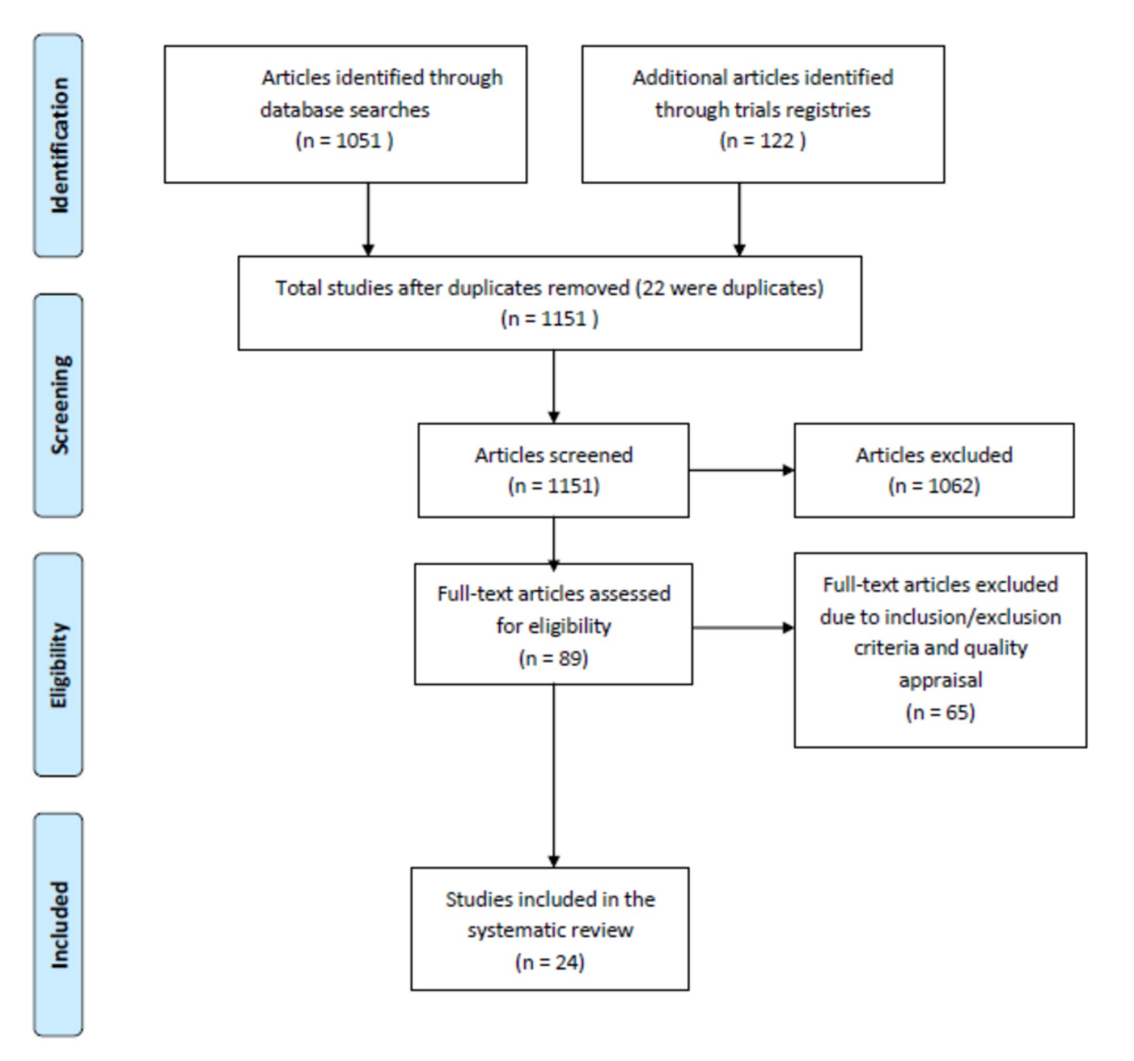
Flowchart of the study selection

*2.1. Literature Search* We identified a total of 1,051 articles from the electronic databases after applying our search strategies and search contents: 210 articles from PubMed, 262 from PubMed Central, 355 from Google Scholar, 166 from Medline, and 58 from ScienceDirect. Additional 122 articles were identified from two trial research registries: 118 from the Cochrane Database Registry and four from the clinicaltrial.gov registry. The sum total of the identified articles was 1,173. Out of these, 111 articles were selected for inclusion and the remaining 1,062 articles were considered irrelevant to the study by the reviewers after a review of their abstracts and were discarded. Twenty-two articles were found to be duplicates and thus removed and the remaining 89 full-text articles were thoroughly reviewed and 56 articles did not satisfy the inclusion and exclusion criteria and thus excluded. Additional nine articles were removed following a quality appraisal. Therefore, only 24 articles were included in the systematic review. A flowchart illustrating the study selection process is depicted in Figure 1.

*2.2. Study Characteristics* The distribution of the included articles from the electronic databases was as follows: 13 studies were obtained from PubMed [13-25]; four from Google Scholar [26-29]; one from PubMed Central [16], five from Cochrane Registry [10,30-33]; and one from clinicaltrial.gov [19]. This is depicted in Table 1.

**Table 1:** Distribution of the included articles from the electronic databases PMC: PubMed Central

Databases	Number of studies	Number of participants
PubMed	13	422
Cochrane Registry	5	105
Google Scholar	4	227
PMC	1	11
Clinicaltrial.gov	1	20
Total	24	785

The majority of the participants in the included study were adults aged more than 50 years (n=512), accounting for more than 65% of the total participants. The distributions of the included studies with respect to the basic demographic information (age, sex, and race); country of study; data characteristics (study design, stroke phase, and stem cell type); intervention characteristics (cell source and route of administration); and time window (time between the onset of stroke and stem cell administration) are depicted in Table 2, Table 3, Table 4, Table 5, and Table 6.

**Table 2:** Basic demographic information

Demographic data	Number of subjects
Age (year)	
Above 50	512
Below 50	273
Sex	
Males	447
Females	338
Race	
White Americans	112
Black Americans	20
Europeans	192
Asians	172
Indians	140
Other	149

**Table 3:** Study countries

Country	Number of studies	Number of subjects
United States	6	147
United Kingdom	5	172
India	2	140
Japan	2	24
South Korea	2	82
Taiwan	2	40
Germany	1	20
Russia	1	20
China	1	6
Cuba	1	5
United States/United Kingdom	1	129
Total	24	785

**Table 4:** Data characteristics

Data characteristics	Number of studies	Number of participants
Study design		
Randomized clinical trial	12	615
Non-randomized trial	12	170
Stroke phase		
Acute	12	324
Sub-acute	4	228
Chronic phase	7	208
Combined acute/sub-acute	1	25
Stem cell type		
Bone marrow mononuclear	6	192
Mesenchymal stem cells	5	148
Hematopoietic stem cells	10	299
Neural stem cells	2	140
Combined neural/mesenchymal	1	6

**Table 5:** Intervention characteristics

Intervention	Number of studies	Number of participants
Source		
Autologous cell	13	389
Allogenic	9	326
Unknown	2	70
Route of administration		
Intravenous	11	442
Subcutaneous	6	206
Intracerebral	4	64
Intra-arterial	3	73

**Table 6:** Time window

Time window	Number of studies	Number of participants
<12 hours	1	20
12-72 hours	5	194
73 hours-3 months	10	393
>3 months	5	155
Unknown	3	23

*2.3. Controlled Studies* Twelve out of the 24 studies were controlled studies, with both intervention and controlled groups. The total number of patients in the intervention group was 313 with 166 male patients and 147 female patients. The total number of patients in the controlled group was 302 patients with 161 male patients and 141 female patients. Seven of these studies used hematopoietic stem cells with almost 90% (n=6) using granulocyte colony-stimulating factor (G-CSF), two studies making use of bone marrow mononuclear cells, two studies utilizing mesenchymal stem cells, and only one study using neural stem cells. Three of the studies combined both stem cell therapy and conventional stroke therapy. In five studies, the controlled group received conventional stroke therapy, in four studies they received placebo, and there was no mention of any therapy given to the controlled group in three studies.

*2.4. Single-Arm Studies* Twelve studies were single-arm types without a comparator group, with total subjects of 170 patients comprising of 120 male patients and 50 female patients. Four of these studies used bone marrow mononuclear cells, four studies utilized mesenchymal stem cells, two studies made use of hematopoietic stem cells, one study used neural stem cells, and one study used umbilical cord blood.

*2.5. Dosing* Seven out of the nine studies that used hematopoietic stem cells utilized G-CSF with the dose range of 1-15 microgram/kg body weight and the majority of them (n=6) were given using the subcutaneous route. The dose range for the bone marrow mononuclear cells used in the six studies was 10-500 million cells with the majority (n=4) given via the intravenous route. Six studies also used mesenchymal stem cells with a dose range of 0.5-150 million cells and the majority (n=5) were given intravenously. The neural stem cells used in two of the studies had a dose range of 2-1,200 million cells delivered via intravenous and intracerebral route. The remaining three studies were separate: CD34+, ALD-401, and umbilical cord blood with maximum doses of 2.42 million cells intra-arterially, 750 million cells intra-arterially, and 15.4 million cells intravenously.

*2.6. Risk of Bias Assessments* Allocation: all the included randomized studies explicitly mentioned that randomization was carried out; however, only seven studies clearly explained the method of the randomization with sequence generation. The allocation was concealed in a majority of these studies with a few patients refusing participation after the allocation. Blinding: 11 studies were blinded; however, the method of blinding differed across the studies. Six studies were double-blinded, involving blinding of both the participants and the research team with 1/6 studies having blinded outcome assessment. Three studies were single-blinded with blinding of the participants only and 1/3 studies blinded outcome assessment. Two studies blinded outcome assessment but the patients and the research teams were not blinded. Therefore, only 4/24 studies effectively blinded outcome assessment. Follow-up: the duration of follow-up was in the range of 6-24 months with only one study having a five-year follow-up duration. In 8/24 studies, all participants were said to have completed the follow-up and therefore included in the final analysis. In another 8/24 studies with a total of 329 participants, some 71 participants were lost to follow-up. In the remaining 8/24 studies, it was not clear whether the participants completed the follow-up or if some were lost to follow-up.

2.7. Outcome Assessment

2.7.1. Primary outcomes: 22/24 studies reported primary outcomes after the follow-up period, generally assessing one or more of the following primary outcomes: neurological impairment measured by NIHSS (from 0-42), disability measured by mRS (from 0-6), and dependency measured by BI (from 0-100). Eleven out of the 22 studies measured all of these primary outcomes, 5/22 studies measured two of these primary outcomes, which were neurologic impairment and disability, 2/22 studies reported disability and dependency, 1/22 study reported neurologic impairment and dependency, and 3/22 studies reported disability only. Virtually, all of these studies reported favorable clinical outcomes across these measured primary outcomes. Sixteen studies reported NIHSS and for the majority of the studies (n=10), the score was in the range of 0-5 with a maximum score of 10, which is a good clinical outcome. Twenty-one studies measured mRS and the majority of the studies (n=14) had mRS of <3 with 5 as the highest reported score, which we also considered as a good clinical outcome. Fourteen studies measured BI score with the majority of the studies (n=8) having BI of >85 with 55 as the lowest recorded BI score. This was also considered a favorable clinical outcome.

2.7.2. Secondary outcome/safety events: the major adverse events reported across the studies were death, infection, hemorrhagic transformation of the infarction, seizures, fever, stroke recurrence, pain, and neoplasm. Death was reported in 9/24 studies and most were not related to the stem cell transplantation; infection was reported in 4/24 studies, hemorrhagic transformation in 5/24 studies, seizures in 3/24 studies, fever in 3/24 studies, stroke recurrence in 2/24 studies, pain in 1/24 study, and neoplasm in 1/24 studies

3. Discussion

This review showed a modest improvement in the clinical outcomes in patients receiving stem cell therapy based on the analysis of the various scales used to measure the clinical outcomes of the pooled studies in this systematic review. However, almost similar findings were found in other studies and the Cochrane Review, where a reduced neurological impairment was found in ischemic stroke patients treated with stem cell transplantation [4,34]. The improvement in the domains of the functional impairment was slightly higher in those with a controlled group than in the single-arm studies; this might be partly due to the presence of less bias in those studies as compared to the single-arm studies and partly due to the larger sample size. This is supported by the findings of some other studies and some systematic reviews, as in the study by Nagpal et al. (2017) in which the safety and feasibility of administering different types of stem cell therapies in stroke patients were reasonably established [6,35].

There was moderate clinical heterogeneity in both subgroups: controlled and single-arm studies. However, it was slightly higher in the controlled group and this could be explained by its larger sample size. Additionally, slightly higher favorable clinical outcomes were observed in those studies where the intravenous route was used and a higher dose of the stem cell was administered with a longer (a minimum of 12 months) follow-up period. The possible explanation could be the easy administration conferred by the intravenous route, which had an early onset of action and much drug bioavailability, provided by the larger dose and the longer follow-up duration to adequately observe the drug effects. Although, an insight can be drawn from this finding that a favorable clinical outcome can be expected when a high dose of stem cells was administered via the intravenous route in a patient with acute or sub-acute stroke, a reliable and acceptable clinical conclusion cannot be drawn from the findings alone.

The adverse events observed in these studies were reassuring, and this has led us to conclude that stem cell transplantation in stroke patients is relatively safe with minimal and manageable side effects. Most of the deaths observed in the studies were due to some underlying comorbidities not generally related to the stem cell intervention. Other adverse effects reported apart from death were infections, hemorrhagic transformation of the infarct, fever, seizures, and pain. No adverse events were reported with respect to the subject recruitment or administration of the therapy, indicating the safety profile of stem cell transplantation. Despite many forms of stem cell therapy, different route of administration, varied ranges of doses, and cell sources in different phases of stroke, our review showed that stem cell transplantation of different kinds via any route in patients with any phase of ischemic stroke is relatively feasible, effective, and has an acceptable safety profile. However, the evidence to support this is not adequate as most of the sample sizes across the studies in both intervention and controlled groups were relatively small and some of the patients in the studies received conventional stroke therapy with few of them having had thrombolytic therapy before the administration of the stem cell. Therefore, it could be difficult to ascribe the improvement in the clinical outcomes to stem cell transplantation alone.

Many studies have demonstrated the benefits of stem cell therapy in stroke patients. In a meta-analysis by Jeong et al. (2014), stem cell therapy involving bone marrow mononuclear cells, mesenchymal and fetal stem cells in stroke patients was shown to be effective in improving many domains of clinical outcomes [20]. Although the dose-response relationship was not clearly demonstrated in this systematic review, some studies have suggested a positive correlation between the dose of the transplanted stem cells and the improvement in functional outcomes [21,36]. In our review, the data showed a slight trend towards better efficacy with the intravenous route as compared to other routes, despite the lack of supportive evidence from clinical trials. However, considering the risks associated with other routes like the risk of embolism in the intra-arterial route and the benefits of the intravenous route like the ease of administration, it would be reasonable to argue that the intravenous route is better than other routes in stem cell transplantation, which is clearly supported by many preclinical trials and meta-analyses of preclinical studies [9]. Despite evidence of reasonable safety and efficacy of stem cell therapy in stroke patients, there are many concerns and gaps in providing preclinical stem cell research to patients with stroke. Therefore, many more randomized and non-randomized studies are needed to address this issue. 

This systematic review has both scientific and clinical benefits. Scientifically, the paper will help the scientific community and future researchers as it involved an exhaustive search conducted via major databases in an attempt to gather all the available information on stem cell transplantation in patients with brain ischemia. Clinically, the paper provides data on and supports the previous body of evidence regarding the feasibility, efficacy, and safety of stem cell transplantation in stroke patients, thereby proving the clinical benefits of the therapeutic method in these cohorts of patients. This understanding will pave way for future researchers to develop interest and invest their time and energy to develop novel stem cell therapeutic strategies.

Our study is not free of limitations. The efficacy and adverse events could not be specifically examined in line with individual stem cells and the specific type of therapy, stroke phase, and timing of the therapy. The overall safety profile and efficacy have mostly been generalized. Hence, specific studies pertaining to the type, dose, duration and route of therapy, and nature and phase of stroke would be more beneficial in the future.

## Conclusions

Stem cell therapies in stroke patients are potentially effective therapeutic strategies for the promotion of recovery in patients with ischemic stroke. Some studies have shown that stem cell therapies have profoundly enhanced the clinical outcomes in patients with brain ischemia, leading to increased attention among scholars towards the potential benefits of stem cell therapies in patients with ischemic stroke. In this systematic review, we demonstrated that stem cell therapies in patients with brain ischemia via any route are essentially safe, effective, and feasible. However, more studies are needed to develop protocols for stem cell transplantation with regard to the cell type, stroke type, stroke phase, route of administration, cell dose, cell source, time window, patient demographic (age, sex, race), and the possibilities of combination therapy. In essence, more studies are earnestly required to promote the clinical application of stem cell transplantation in patients with stroke, particularly ischemic stroke.
